# Body Composition and Physical Fitness: Does This Relationship Change in 4 Years in Young Adults?

**DOI:** 10.3390/ijerph19031579

**Published:** 2022-01-29

**Authors:** Maciej Kochman, Wojciech Kasperek, Agnieszka Guzik, Mariusz Drużbicki

**Affiliations:** Physiotherapy Department, Institute of Health Sciences, College of Medical Sciences, University of Rzeszów, Marszałkowska 24, 35-215 Rzeszów, Poland; wkasperek@ur.edu.pl (W.K.); aguzik@ur.edu.pl (A.G.); mdruzbicki@ur.edu.pl (M.D.)

**Keywords:** physical fitness, physical activity, body composition, BMI

## Abstract

(1) Background: There are few studies investigating the relationship between physical fitness and body composition in young adults and, to our knowledge, there are no such reports focusing specifically on physiotherapy students. This observational study aimed to assess the relationship between physical fitness and selected anthropometric measurements as well as body composition in Polish students in the first year and in the final year of a university course in physiotherapy. (2) Methods: A group of 100 randomly selected individuals were recruited among first- and fifth-year students of physiotherapy (50% women), who were assigned to two groups: A (aged 19 years) and B (aged 23 years). Body composition was assessed using a Tanita TBF-300 Analyzer, and physical fitness was measured using Zuchora’s Physical Fitness Index. (3) Results: A higher level of general fitness was identified in students from Group B (*p* = 0.0261), and lower values of the fat mass index was found in Group A (*p* = 0.0441). Group A was found with correlations between general level of physical fitness and the following indexes: fat% (*R* = −0.4; *p* = 0.0018), FM (*R* = −0.3; *p* = 0.0310), FFM (*R* = 0.3; *p* = 0.0229) and TBW (*R* = 0.4; *p* = 0.0024), whereas Group B was found with correlations between general physical fitness and BMI (*R* = 0.3; *p* = 0.0308) as well as FM index (*R* = 0.3; *p* = 0.0431). (4) Conclusions: The findings show significant differences between the groups in physical fitness, body composition and selected anthropometric measurements. Older students presented higher level of general fitness, whereas younger students were found with a lower mean value of fat index.

## 1. Introduction

Insufficient levels of physical activity associated with sedentary lifestyles as well as bad eating habits and high-calorie diets lead to obesity and other diseases of affluence such as diabetes, atherogenic hyperlipidaemia and arterial hypertension. Co-occurrence of these conditions may lead to metabolic syndrome or adverse cardiovascular incidents as well as premature death [1]. Regular physical activity leads to positive changes in the human body, making it possible to prevent diseases of affluence, including obesity and cardiovascular problems [2]; reduce excessive adipose tissue [3]; improve strength, muscular endurance and motor coordination [4]; reduce the risk of dementia and depression [5,6,7]; improve bone mineralisation [8]; strengthen the immune system [9]; improve efficiency of the respiratory processes and increase blood flow rate, resulting in more effective supply of oxygen and nutrients to the organs and tissues [10]. Exercise stimulates and improves functioning of the human body by increasing or maintaining its physical efficiency and capacities [10,11]. 

Physical fitness is an important determinant of health, and it is associated not only with the locomotor system, but also with the overall biological functioning of the body [11]. It is assumed that physical fitness is related to both good performance in motor exercises and effective work of the specific organs and systems. It is distinct to each individual, it is linked to specific functions and predispositions of each person, and it is manifested in selected motor effects. Scores in fitness tests are related to sex and age as well as body mass and to more detailed parameters such as body fat, muscle mass and body water percentage [12]. 

Human body mass is sensitive to changes resulting from energy balance, which is the difference between the energy supplied with nutrition and the energy expenditure needed for the functioning of the body and additional physical activity [13]. A positive energy balance leads to excessive accumulation of adipose tissue in the body, resulting in overweight or obesity [14]. Assessment of body tissue parameters, such as fat-free body mass and body fat percentage as well as body water percentage, is important for a variety of reasons. Most frequently, research is conducted to determine appropriate diet, to investigate the effects of exercise, to select appropriate training (including physiotherapy) and to establish developmental norms. The assessment of body composition is conducted, for example, with the use of anthropometric measurements and bioelectrical impedance analysis [15,16,17].

From a scientific point of view, the motivation to undertake this research was the fact that weight disorders as well as insufficient levels of physical activity in young adults are nowadays a significant health and social problem in most developed countries [18] including Poland [19]. Numerous reports indicate that weight disorders and the sedentary lifestyles of young adults lead to many diseases risk factors such as hypertension [20], hyperlipidaemia [21], type 2 diabetes [22] or cancer [23].

In the related literature, there are many reports from research investigating relationships between physical activity and body composition; however, they mainly focus on populations of children and adolescents as well as individuals with obesity [24,25]. On the other hand, there are few studies investigating this relationship in specific populations of young adults [26,27,28] and, to the best of our knowledge, no studies have examined the relationship in individuals starting and approaching the end of the university course in physiotherapy. Given this, we conducted the present observational study in order to determine whether: (1) there were differences in the level of fitness and body composition between first- and fifth-year students of physiotherapy; (2) there was a relationship between body composition, selected anthropometric measurements and general fitness in the studied groups.

## 2. Materials and Methods

### 2.1. Study Participants

For this observational study, we recruited a random group of 100 healthy volunteers (first- and fifth-year physiotherapy students). Participants were divided into two groups: group A (aged 19 years; first-year students only; 50% female) and group B (aged 23 years; fifth-year students only; 50% female). The two groups did not differ significantly in body height, weight or BMI. The study groups’ characteristics are shown in Table 1. 

To be included in the study, participants had to be aged 19 years and be first-year physiotherapy students or 23 years and fifth-year physiotherapy students, free of any injury, pain or other conditions suggesting that the participant would probably not be able to perform physical exercise safely for at least 6 months.

The minimum size of the sample was calculated taking into account the number of individuals studying physiotherapy. A fraction size of 0.8 was applied, with a maximum error of 7%, and as a result a sample size of 99 people was determined. One hundred individuals were qualified for the study.

### 2.2. Study Design

The protocol of this prospective observational study was approved by the Ethics Committee of the Rzeszow University (ref. no. 10/2/2015) and examinations were carried out in compliance with the Declaration of Helsinki. Examinations and tests were conducted in the morning. Before the start, each participant was informed about the examination stages and procedures. Subsequently, they gave their written informed consent to participate in this examination. Body composition was measured using a Tanita TBF-300 Analyzer (TANITA, Middlesex, UK), and physical fitness was measured using Zuchora’s Physical Fitness Index.

### 2.3. Anthropometrics and Body Composition Measures

**Figure 1:**
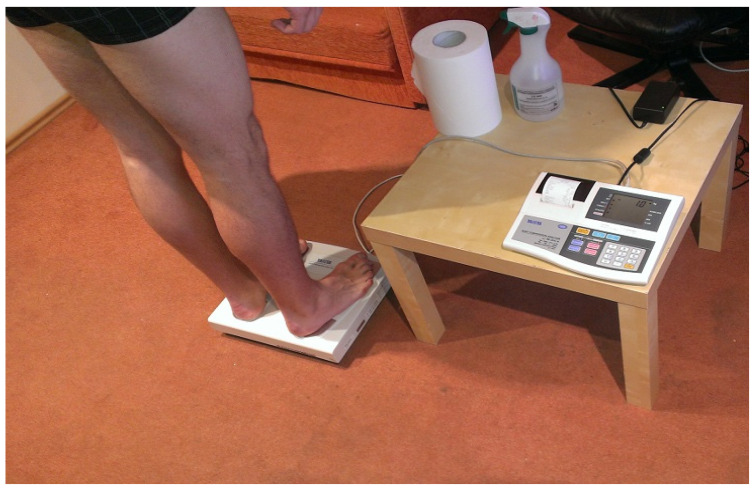
Tanita TBF-300 measurement method.

Measurements of body composition were performed using a Tanita TBF-300 Analyzer, which applies a bioelectrical impedance method. This means that the device measures the total electrical resistance of the body by applying an electric current of a specific frequency and intensity. The examination is painless and non-invasive, and the procedure does not take long. It can be applied to subjects of both sexes and of all ages. This device has demonstrated strong evidence of concurrent validity (r = 0.94; *p* < 0.001) comparing with the “criterion standard” of dual-energy X-ray absorptiometry (DEXA) for %BF [29]. Before the measurement of body composition, each participant was informed about the method applied during the examination. All the measurements were carried out during morning hours by the same physiotherapist trained in the operation of the device. The subjects were asked to take off their outer clothing, shoes and socks at a specified place and then to stand on the plate of the analyser. The participants were tested on an empty stomach, and they were asked to stand upright with their body weight fully supported on both legs [29,30]. The plate of the analyser was disinfected before each subsequent examination. Figure 1 shows how the Tanita TBF-300 measurement was performed.

**Figure 2:**
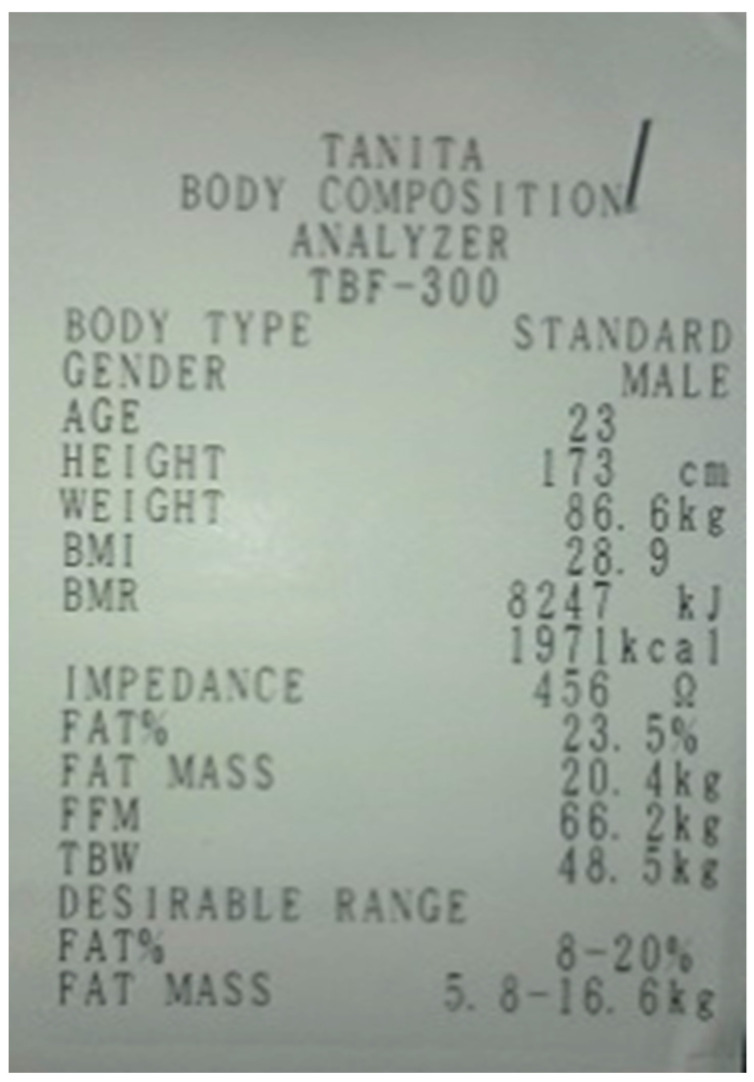
Sample Tanita TBF-300 printout.

Based on body composition analysis, the following were determined: overall body weight with an accuracy of 0.1 kg, BMI, fat% (body fat percentage), fat mass, FFM (fat-free mass) and TBW (total body water) [30,31]. Figure 2 presents a sample printout from the Tanita TBF-300 device.

### 2.4. Physical Fitness Measures 

Assessment of physical fitness was performed using the Physical Fitness Index proposed by Krzysztof Zuchora. The motor tasks are planned in such a way that they can be performed by anyone, in any conditions and without unnecessary devices. The total score obtained by the subject in all the trials is a measure of his/her capacities. As a result, the score can be analysed by reference to the norms defined for the specific age groups relative to sex. Furthermore, it can also be used to make comparisons with the person’s physical fitness at different times of life and with the achievements of other people [32,33]. The index comprises 6 trials: speed test, jumping ability test, arm strength test, flexibility test, endurance test and abdominal muscle test [34,35]. A detailed description of all trials is included in Appendix A.

Prior to the test, each subject performed warm-up exercises for 10 min to prepare the body for the physical strain [32]. All the fitness tests were carried out by the same physiotherapist experienced in performing fitness tests. Detailed criteria for assessment in the specific tests are shown in Appendix B.

### 2.5. Statistical Analysis

The collected data were processed using the software Statistica 13.1. Nonparametric tests were applied in the analysis because of the failure to meet the basic assumptions of parametric tests, i.e., distribution of the data corresponding to normal distribution and homogeneity of variance. The normality of distributions was examined using the Shapiro–Wilk W test, and the homogeneity of variance was assessed with the Levene test. Statistical significance was assumed if *p* < 0.05. The nonparametric Mann–Whitney U test was applied to compare quantitative data corresponding to anthropometric measures, body composition and physical fitness. Relationships between overall physical fitness and selected anthropometric measures as well as body composition were assessed using Spearman’s rank correlation coefficient.

## 3. Results

Analysis of the scores in the physical fitness test showed statistically significant differences between Groups A and B in endurance, standing jump and abdominal muscle strength as well as general level of physical fitness. Assessment of endurance showed that subjects in Group A on average achieved a higher score, compared to subjects in Group B (4.98 ± 0.89 vs. 4.62 ± 0.85; *p* = 0.0271). The standing jump test identified very high statistically significant differences at a level of *p* < 0.001 between Groups A and B. Better results were achieved by students aged 23 years (4.28 ± 0.99 vs. 3.26 ± 1.24, *p* = 0.0001). In abdominal muscle strength tests, students in Group A on average achieved poorer scores compared to students in Group B (3.30 ± 0.91 vs. 4.50 ± 0.97; *p* < 0.001). A total score achieved in the six physical fitness tests was calculated and taken into account in determining the mean result for general fitness of the subjects. Higher general physical fitness was observed in students from the B group (26.34 ± 3.64 vs. 24.54 ± 3.93; *p* = 0.0261). 

Analysis of body composition parameters showed statistically significant differences only in the value of the fat mass index. The mean value of this index was lower in younger students (10.99 ± 6.0 vs. 13.40 ± 8.02; *p* = 0.0441). Comparison of physical fitness level and body composition is shown in Table 2.

At the next stage, we examined the relationships between the students’ general fitness level and selected measures, such as body height and weight and BMI as well as impedance, fat%, fat mass, fat-free mass and total body water. Because of the quantitative nature of the data, the Spearman rank correlation coefficient was used in the analyses. The relationships between the students’ physical fitness and selected anthropomorphic measures are presented for the entire population studied, relative to age (separately for Group A and Group B). Correlation of general fitness vs. anthropometrics and body mass composition is shown in Table 3.

For the entire population studied, a positive statistically highly significant yet weak or very weak correlations were found between general physical fitness and body weight (*R* = 0.2; *p* = 0.0262) and BMI (*R* = 0.2; *p* = 0.0278) as well as fat-free mass (*R* = 0.3; *p* = 0.0051) and total body water (*R* = 0.3; *p* = 0.0007). This means that higher body weight, BMI, fat-free mass and total body water corresponded to higher scores achieved by the subjects in the Zuchora Fitness Test. As regards the impedance index, the findings showed a negative statistically significant but very weak correlation (*R* = −0.2; *p* = 0.0283), which means that a higher impedance index corresponded to lower scores achieved by the subjects in the Zuchora Fitness Test.

For the subjects in Group A (19 years of age), negative statistically highly significant but weak correlation was identified between general physical fitness level and the indexes fat% (*R* = −0.4; *p* = 0.0018) as well as fat mass (*R* = −0.3; *p* = 0.0310). This means that higher values of fat% and fat mass indexes in the group of 19 year old subjects corresponded to lower scores in the Zuchora Fitness Test. Positive statistically highly significant yet weak correlation was found between general physical fitness and the values of fat-free mass (*R* = 0.3; *p* = 0.0229) and total body water (*R* = 0.4; *p* = 0.0024). This means that higher values of the fat-free mass index in the 19 year olds corresponded to a higher score in the Zuchora Fitness Test.

As regards the subjects in Group B (23 years of age), positive statistically highly significant yet weak correlation was only found between general physical fitness and BMI (*R* = 0.3; *p* = 0.0308) as well as fat mass index (*R* = 0.3; *p* = 0.0431). This means that higher values of BMI and fat mass in the 23 years olds corresponded to a higher score in the Zuchora Fitness Test.

## 4. Discussion

In our study, we found significant differences in endurance, standing forward jump and abdominal muscle strength tests as well as the general level of physical fitness. The scores reflecting general physical fitness and those identified in the specific tests, with an exception for the endurance test, were higher in students in the final year of the physiotherapy course. Better physical fitness in this group may be linked to the curriculum of the course mainly comprising classes in which students learn about various forms of exercise to be applied in future clinical work with patients. The classes and lectures may have increased the students’ awareness that active lifestyles and a high level of physical fitness are helpful in maintaining good health status as a result of which the subject may have been encouraged to take up additional physical activity in their personal lives.

Human body mass is sensitive to changes resulting from a variety of factors; one of these is the level of physical activity. Excessively accumulated adipose tissue, just like insufficient activity and physical fitness, may lead to various diseases of affluence and to various health problems [36,37]. Like in the case of insufficient physical fitness, the causes of pathologically excessive adipose tissue include sedentary lifestyles, passive leisure activities, high-calorie diets and unhealthy eating habits [37]. Assessment of body mass makes it possible to determine adequate diet and program for physical training and physiotherapy and to evaluate the effectiveness of interventions [38]. The present study shows that the content of adipose tissue in the two groups was similar to the generally accepted norms specified for the relevant age group [39]; however, in the older group the value was significantly higher than in the younger subjects. The higher body fat index in the older group may be linked to the global trend and obesity epidemic and to the fact that it significantly and positively correlates with age [40]. Supplementary information concerning potentially impaired nutritional status in the population studied would be provided by additional anthropometric measurements such as waist and hip circumference or waist-to-hip ratio and waist-to-height ratio [41]. 

The present study also investigated possible correlations between general physical fitness and selected anthropometric measurements as well as body composition. Analysis of the results showed that only some of the anthropometric measures and body composition indexes may be related to general physical fitness. In the case of the entire population studied, better physical fitness was correlated with a lower impedance index and higher body weight as well as higher BMI, fat-free mass and total body water. Our findings are interesting due to the fact that in the related literature, there are many reports suggesting that higher body weight and higher BMI are strongly correlated to a lower level of activity and poorer physical fitness [42,43]. It should be emphasised, however, that although body weight and BMI can be measured easily and quickly, the two methods present a drawback since they do not make it possible to calculate the ratio of body fat content to fat-free body mass [39,41]. It can be hypothesised that the relationship between better physical fitness and higher body weight as well as BMI in the subjects examined in the present study may be associated with more developed muscle mass, which is reflected by the higher values of fat-free mass and total body water; this has also been shown in other studies [44,45]. The correlations between the general fitness of physiotherapy students and the selected anthropometric measurements as well as body composition indexes in the present study were also investigated relative to the age groups. In the younger group, comprising first-year students, better physical fitness was correlated to lower fat% and fat mass indexes and to higher fat-free mass and total body water. In the older group, comprising fifth-years students, such correlation was found only with higher BMI and fat mass. The differences in these relationships may be explained with the fact that the adipose tissue content was lower in younger students, while in the case of older students, a greater content of adipose tissue may have been compensated by more activities involving physical exercise performed in the course of study and, possibly, by greater awareness of the beneficial health-promoting effects of regular exercise. 

To recapitulate the above considerations, it should be pointed out that the present findings open new areas for research related to the subject matter, and they may serve as encouragement for further in-depth investigations where university students of other specialisations should be examined in order to determine whether university courses in physiotherapy that incorporate lots of physical exercise produce beneficial effects as regards the students’ physical fitness. Furthermore, the related research should also take into account practicing physiotherapy professionals and should investigate potential changes in physical fitness and the relationship between physical fitness and body composition, for instance during four years after graduation from university. Notwithstanding the above, people generally should be encouraged to follow healthy lifestyles, and undertake various forms of physical activity in order to improve their condition and maintain good health status. Physiotherapists play an important role in this process, as individuals providing rehabilitation and preventive treatments, and they should serve as role models for society. The obtained results may have practical application providing an incentive to develop supplementary physical activity programs tailored to the relevant groups of physiotherapy students, which could be introduced into the study program as its supplement. This fact should be taken into account by designers of study programs for physiotherapy students.

A limitation of the study is linked with the fact that only two age groups (i.e., 19 and 23 year old students) were taken into account. It seems necessary to conduct a controlled study taking into account more age groups of young adults. It would be interesting to see the impact of various factors, such as the subjects’ eating habits or waist circumference, on the methodology. Such analysis could be included in future studies. Future research should also be expanded to include an assessment of the physical activity level and—given the higher content of adipose tissue in the older students—it would be worthwhile to introduce other measures and tools into the study to assess nutritional status and the related risks by assessing waist circumference or conducting nutrition-related interviews. It would also be interesting to assess correlations between body composition and scores in the specific trials of the physical fitness test. Therefore, the presented results should be treated as preliminary and should be followed by a study involving the abovementioned items.

## 5. Conclusions

The comparative assessment of physiotherapy students in the first and fifth year of a university course has shown significant differences between these groups in body composition and physical fitness. Older students presented higher levels of overall fitness, whereas younger students were found with a lower mean value of fat index. The students’ general physical fitness was also shown to be significantly correlated with body composition and selected anthropometric measurements. It is necessary to continue the related research, taking into account university students of other specialisations in order to determine whether or not university courses in physiotherapy, incorporating lots of physical exercise, produce beneficial effects as regards the students’ physical fitness; it would also be a good idea to investigate practicing physiotherapy professionals and assess potential changes in physical fitness and the relationship between physical fitness and body composition over a period of several years following graduation from university.

## Figures and Tables

**Table 1:** Study group characteristics.

*N* = 100			
Variable	Group A (*N* = 50)	Group B (*N* = 50)	*p*
Gender (m/f)	25/25	25/25	
	Mean (SD)	Mean (SD)	
Age	19	23	
Height (cm)	1.71 (0.09)	1.71 (0.09)	0.6023
Body mass (kg)	64 (11.88)	66.45 (16.33)	0.7907
BMI (kg/m^2^)	21.43 (2.30)	22.47 (3.93)	0.2836

*N*—number of participants; SD—standard deviation; m—males; f—females; BMI—body mass index.

**Table 2:** Physical fitness and body composition measurements.

*N* = 100			
Variable	Group A (*N* = 50)	Group B (*N* = 50)	*p*
	Mean (SD)	Mean (SD)	
Physical fitness			
Endurance	4.98 (0.89)	4.62 (0.85)	0.0271
Speed	4.96 (1.07)	4.72 (0.81)	0.1055
Flexibility	4.50 (1.54)	4.22 (1.37)	0.2461
Jumping ability	3.26 (1.24)	4.28 (0.99)	0.0001
AMS	3.30 (0.91)	4.50 (0.97)	0.0000
Arm strength	3.58 (1.09)	4.00 (1.12)	0.1026
GPF	24.54 (3.93)	26.34 (3.64)	0.0261
Body composition			
Impedance (Ω)	514.92 (63.32)	537.08 (62.91)	0.0925
Fat%	16.91 (7.35)	19.29 (7.42)	0.0867
Fat mass (kg)	10.99 (6.00)	13.40 (8.02)	0.0441
Fat-free mass (kg)	53.02 (10.15)	53.05 (11.59)	0.5931
TBW (kg)	38.01 (8.13)	38.83 (8.48)	0.9204

*N*—number of subjects; SD—standard deviation; AMS—abdominal muscle strength; GPF—general physical fitness; TBW—total body water.

**Table 3:** Correlation of general physical fitness vs. anthropometrics and body mass composition.

*N* = 100	General Physical Fitness					
Variable	Group A (*N* = 50)		Group B (*N* = 50)		Total (*N* = 100)	
	*R*	*p*	*R*	*p*	*R*	*p*
Height	0.2	0.0967	0.2	0.2931	0.2	0.0606
Body mass	0.1	0.3366	0.3	0.0576	0.2	0.0262
BMI	0.1	0.6876	0.3	0.0308	0.2	0.0278
Impedance	−0.2	0.0859	−0.3	0.0595	−0.2	0.0283
Fat%	−0.4	0.0018	0.1	0.4130	−0.2	0.1229
Fat mass	−0.3	0.0310	0.3	0.0431	0.0	0.8153
Fat-free mass	0.3	0.0229	0.3	0.0728	0.3	0.0051
Total body water	0.4	0.0024	0.3	0.0723	0.3	0.0007

*N*—number of subjects.

## Data Availability

The datasets used and/or analysed during the current study are available from the corresponding author on reasonable request.

## Appendix A

Description of Zuchora’s Physical Fitness Index Trials:

- Speed test (running in place, raising knees high with each step, and clapping one’s hands under the raised knee; trial duration 10 s; the score is the number of claps);
- Jumping ability test (standing forward jump, the length measured with one’s own feet; the score is the distance corresponding to the number of feet);
- Arm strength test (hanging from a bar, using various methods of increasing difficulty for the specific grades; the result is the duration of hanging or pulling-up activity);
- Flexibility test (standing forward bend; the score corresponds to the depth of the bend);
- Endurance test (running in place as long as possible; the result corresponds to the duration of performance);
- Abdominal muscle strength test (horizontal scissor kicks while lying on one’s back; the result corresponds to the duration of performance).

## Appendix B

**Table A1:** Assessment norms of the Physical Fitness Index by Zuchora.

Trial	G	General Physical Fitness					
		Minimal 1 Point	Poor 2 Points	Good 3 Points	Very Good 4 Points	High 5 Points	Excellent 6 Points
Speed (claps)	F	12	16	20	25	30	35
	M	15	20	25	30	35	40
Jumping ability (feet)	F M	5	6	7	8	9	10
Arm strength	F	Extended two-arm hang for 3 s.	Extended two-arm hang for 10 s.	Extended one-arm hang for 3 s.	Extended one-arm hang for 10 s	Pulling up on a bar for 3 s	Pulling up on a bar for 10 s.
	M	Extended two-arm hang for 10 s.	Extended one-arm hang for 10 s.	Pulling up on a bar for 3 s	Pulling up on a bar for 10 s.	Pulling up on a bar, letting one arm down and enduring for 10 s.	Pulling up on a bar, letting one arm down and enduring for 10 s. Change of arm.
Flexibility	F M	Grabbing one’s ankles with both hands.	Touching one’s feet with fingers.	Touching the ground with fingers.	Touching the ground with all the fingers.	Touching the ground with hand palms.	Touching knees with one’s head.
AMS	F	10 s	30 s	1 min	1.5 min	2 min	3 min
	M	30 s	1 min	1.5 min	2 min	3 min	4 min
Endurance (min)	F	1	3	6	10	15	20
	M	2	5	10	15	20	30

G—gender; F—females; M—males; AMS—abdominal muscle strength.
